# Spectral Unmixing‐Based Reaction Monitoring of Transformations between Nucleosides and Nucleobases†

**DOI:** 10.1002/cbic.202000204

**Published:** 2020-06-18

**Authors:** Felix Kaspar, Robert T. Giessmann, Sarah Westarp, Katja F. Hellendahl, Niels Krausch, Isabel Thiele, Miriam C. Walczak, Peter Neubauer, Anke Wagner

**Affiliations:** ^1^ Institute of Biotechnology, Chair of Bioprocess Engineering Technische Universität Berlin ACK 24 Ackerstraße 76 13355 Berlin Germany; ^2^ BioNukleo GmbH Ackerstraße 76 13355 Berlin Germany

**Keywords:** nucleobases, nucleoside phosphorylase, nucleosides, spectral unmixing, UV/Vis spectroscopy

## Abstract

The increased interest in (enzymatic) transformations between nucleosides and nucleobases has demanded the development of efficient analytical tools. In this report, we present an update and extension of our recently described method for monitoring these reactions by spectral unmixing. The presented method uses differences in the UV absorption spectra of nucleosides and nucleobases after alkaline quenching to derive their ratio based on spectral shape by fitting normalized reference spectra. It is applicable to a broad compound spectrum comprising more than 35 examples, offers HPLC‐like accuracy, ease of handling and significant reductions in both cost and data acquisition time compared to other methods. This contribution details the principle of monitoring reactions by spectral unmixing, gives recommendations regarding solutions to common problems and applications that necessitate special sample treatment. We provide software, workflows and reference spectra that facilitate the straightforward and versatile application of the method.

## Graphical abstract

**The bases of life** and their analogues display UV absorption spectra that differ from those of their respective nucleosides under alkaline conditions. This enables robust, versatile and fast analysis of reactions between these species.

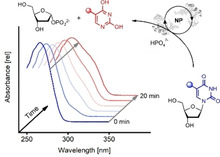

Nucleoside‐altering enzymes harbor significant potential for the synthesis of nucleoside analogues. Nucleoside phosphorylases (NPs), for instance, catalyze the reversible phosphorolytic cleavage of nucleosides into the corresponding free nucleobase and pentose‐1‐phosphate (Scheme 1) and are widely applied for the preparation of modified nucleosides.^1^, ^2^, ^3^, ^4^, ^5^, ^6^, ^7^, ^8^, ^9^, ^10^, ^11^ Consequently, their kinetic and thermodynamic characterization has attracted increased interest and demanded the development of efficient analytical tools.^12^, ^13^

**Scheme 1:**
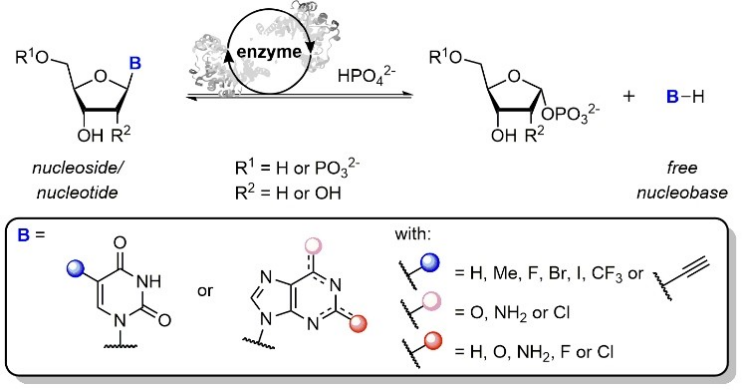
Nucleoside/nucleotide phosphorolysis of pyrimidine or purine species. With the exceptions of cytosine and 1,2,4‐triazole‐3‐carboxamide, all nucleobases featured in this report are described.

Recently, we reported a UV/Vis spectroscopy‐based method for the monitoring of these reactions that largely eliminated the need for HPLC.^14^ For this method, we employed spectral unmixing to derive nucleoside/nucleobase ratios from experimental UV absorption spectra based on suitable reference spectra. Implemented into the workflow of a high‐throughput assay, this methodology facilitated a >20‐fold reduction of data acquisition time and a roughly fivefold decrease in cost compared to conventional HPLC, while maintaining very comparable accuracy and excellent reproducibility. Unlike other non‐HPLC‐based methods,^15^, ^16^, ^17^, ^18^, ^19^, ^20^, ^21^, ^22^, ^23^, ^24^, ^25^, ^26^, ^27^ our approach offers a uniquely broad substrate spectrum, including all natural and several examples of modified nucleosides, as well as high adaptability and the straightforward application to any substrate without the need for laborious method development.

Following the initial report of our method, it has found wide‐spread use in our laboratory and was successfully applied to several projects. Most notably, previous spectral characterization of a range of nucleoside substrates and their corresponding nucleobases enabled the investigation of the thermodynamic reaction control of nucleoside phosphorolysis.^12^ Here we were able to measure slight temperature‐induced changes of reaction equilibria of the phosphorolysis of 24 nucleosides that allowed convenient experimental access to thermodynamic properties of those reactions. Knowledge of the UV absorption spectra of nucleosides and nucleobases also enabled qualitative reaction monitoring of nucleoside transglycosylations to determine the time to equilibrium and reduce sampling effort.^11^ Further work to employ our method for the kinetic characterization of several NPs across their broad working space to probe the limits of their tolerance to harsh reaction conditions is currently underway.^28^ In addition, this method has greatly aided our efforts to explore the substrate spectra of other nucleobase‐cleaving enzymes,^29^ empowered screening projects^30^ and overall alleviated our dependence on HPLC.^31^ Ultimately, these examples showcase the remarkable potential of our spectral unmixing‐based method for high sample throughput and efficient monitoring of nucleobase cleavage reactions.

In this update we expand the scope of established substrates, share our experience and recommendations regarding solutions to common problems and describe some examples of alternative uses of the original method that necessitate deviation from the previously reported protocol. This contribution highlights the utility of spectral unmixing for the monitoring and analysis of (enzymatic) nucleobase cleavage reactions and will prove helpful to all current and future users of our previously published method.^14^

## The principle of reaction monitoring by spectral unmixing

Spectral unmixing in this case describes the concept of linear combination of absorption spectra that can be traced back to its individual components. In this sense, any mixture of two (or more) compounds with known absorption spectra can be deconvoluted into its constituents if appropriate reference spectra are available.^32^

**Figure 1:**
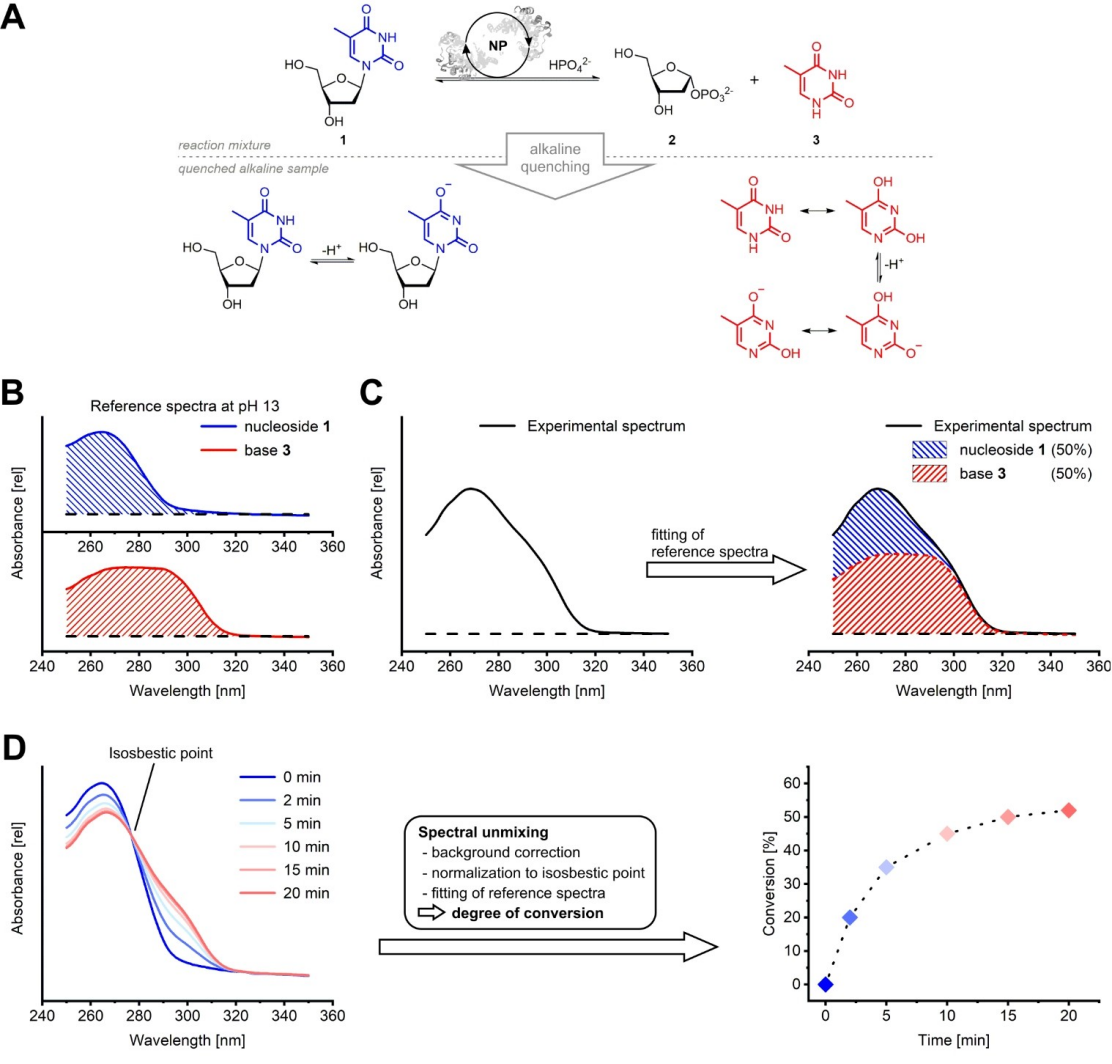
The principle of spectral unmixing‐based reaction monitoring. A) Enzymatic phosphorolysis of thymidine (**1**) into 2‐deoxyribose‐1‐phosphate (**2**) and the free nucleobase **3** as well as deprotonation after alkaline quenching. Representative resonance structures are shown. B) The substrate **1** and product **3** of the reaction have markedly different UV absorption spectra under alkaline conditions. C) The spectra of **1** (blue) and **3** (red) can be fitted to an experimental spectrum (black line) obtained during a reaction to derive the individual contributions of both species to the observed spectrum (hashed areas). D) Unmixing of multiple experimental spectra obtained during a reaction (left) enables reaction monitoring by deriving the degree of conversion at every sampled timepoint (right). Spectral unmixing of nucleoside transformations generally includes background correction, normalization to the isosbestic point of base cleavage, and fitting of the respective reference spectra. The spectra and conversions presented in this figure serve an illustrative purpose and were generated from the reference spectra of **1** and **3**, as described in the externally hosted Supporting Information.^36^ Typical reaction conditions include a nucleoside concentration of 2 mM, 10 mM phosphate, 50 mM buffer of choice and 10 μg⋅mL^−1^ NP in a total volume of 500 μL.^12^

Our method for monitoring of nucleobase cleavage reactions employs this concept by deriving nucleoside/nucleobase ratios from experimental spectra recorded after alkaline dilution of samples from a reaction mixture.^14^ Under alkaline conditions the UV absorption spectra of nucleosides and nucleobases (Figure 1A) differ sufficiently to allow discrimination (Figure 1B).^33^, ^34^, ^35^ Therefore, previously recorded reference spectra can be fitted to a background‐corrected experimental spectrum (Figure 1C) to determine the contribution and ratio of its individual constituents (namely substrate and product of the reaction). This approach allows for efficient reaction monitoring when multiple UV absorption spectra from a given reaction are available and can be deconvoluted into their individual components to derive the respective degree of conversion (Figure 1D). Conveniently, nearly all nucleoside‐nucleobase pairs display an isosbestic point of base cleavage that allows for normalization to correct for differences in signal intensity which in turn eliminates potential errors from pipetting inaccuracy. At the isosbestic point, the nucleoside and nucleobase in question possess the same extinction coefficient which manifests itself as a constant signal intensity at this wavelength throughout a reaction (see Figure 1D for the pair of **1** and **3**).

In this workflow, alkaline dilution of the sample serves a threefold purpose. This step simultaneously terminates the reaction by denaturing the enzyme, adjusts the concentration of the analytes and regulates the pH value of the sample to achieve deprotonation and spectral shifting of the UV absorption spectra. The suitable degree of alkaline dilution as well as the concentration of the base used for dilution (in our case aqueous NaOH) varies between different nucleoside‐nucleobase pairs, since the extinction coefficients and the pH range for stable and reproducible spectra needed for analysis differ. For example, purine nucleosides generally display a stronger UV absorption than pyrimidine nucleosides, which requires smaller sampling volumes to achieve the same peak signal intensity for these substrates.

Although we mainly discuss nucleobase cleavage (e. g., by nucleoside phosphorolysis) in this report, spectral unmixing‐based reaction monitoring can also be applied to observe the reverse reaction. The same principles, strategies and challenges considered below for phosphorolysis reactions also pertain to the corresponding transformations in the glycosylation direction.

## Updated list of established substrates

Extending our previously reported list of 20 nucleosides,^14^ we herein present the spectral characteristics of 38 substrates (Table 1). Reference spectra for all compounds and their bases listed in Table 1 are freely available from the externally hosted supplementary material.^36^ The updated list of established substrates now includes several modified purine nucleosides (**22**–**27** and **33**–**36**) and highly modified pyrimidines such as 5‐trifluoromethyluridine (**15**). Notably, we also characterized some 5’‐phosphorlyated nucleotides and found their spectral properties to be essentially identical to their respective non‐phosphorylated counterparts, conveniently allowing the reaction monitoring for these substrates without the need for additional reference spectra or method development.

**Table 1:** Spectral properties of nucleosides, nucleotides, and their bases under alkaline conditions.

	Compound^[a]^	pH^[b]^	*λ*_max_ nucleoside/nucleotide [nm]	*λ*_max_ nucleobase [nm]	Isosbestic point of base cleavage [nm]	Spectral extension [nm]
Pyrimidines	uridine (**4**)^[c]^	13	262	281	271	310
	2’‐deoxyuridine (**5**)^[c]^	13	262	281	272	310
	5‐methyluridine (**6**)^[c]^	13	267	290	277	320
	thymidine (**1**)^[c]^	13	266	290	278	320
	5‐fluorouridine (**7**)^[c]^	13.3	269	281	282	325
	2’‐deoxy‐5‐fluorouridine (**8**)^[c]^	13.3	268	281	280	325
	5‐bromouridine (**9**)^[c]^	13	276	290	283	330
	5‐bromo‐2’‐deoxyuridine (**10**)^[c]^	13	275	290	282	330
	5‐iodouridine (**11**)^[c]^	13.3	281	291	283	340
	2’‐deoxy‐5‐iodouridine (**12**)^[c]^	13.3	279	291	282	340
	5‐ethynyluridine (**13**)^[c]^	13.3	285	298	262, 288	340
	2’‐deoxy‐5‐ethynyluridine (**14**)^[c]^	13.3	284	298	262, 288	340
	5‐trifluoromethyluridine (**15**)	10	259	279	267	310
	cytidine (**16**)^[c]^	13.7	271	281	271	310
	2’‐deoxycytidine (**17**)^[c]^	13.7	271	281	271	310
	uridine‐5’‐monophosphate (**18**)	13	262	281	271	310
	cytidine‐5’‐monophosphate (**19**)	13.7	271	281	271	310
Purines	adenosine (**20**)^[c]^	13	259	268	267	310
	2’‐deoxyadenosine (**21**)^[c]^	13	259	268	267	310
	2‐fluoroadenosine (**22**)	13	260	268	271	310
	2’‐deoxy‐2‐fluoroadenosine (**23**)	13	260	268	271	310
	2‐chloroadenosine (**24**)	13	264	271	271	310
	2‐chloro‐2’‐deoxyadenosine (**25**)	13	264	271	271	310
	2‐aminoadenosine (**26**)	13	279	284	285	320
	2‐amino‐2’‐deoxyadenosine (**27**)	13	279	284	285	320
	guanosine (**28**)^[c]^	13	264	273	279	310
	2’‐deoxyguanosine (**29**)^[c]^	13	264	273	279	310
	inosine (**30**)^[c]^	13	252	262	263	320
	2’‐deoxyinosine (**31**)^[c]^	13	252	262	263	320
	xanthosine (**32**)	13.3	276	282	276	320
	2,6‐dichloropurine riboside (**33**)	9	274	279	278	310
	2,6‐dichloro 2’‐deoxyriboside (**34**)	9	274	279	278	310
	6‐chloro‐2‐fluoropurine riboside (**35**)	9	269	273	271	310
	6‐chloro‐2‐fluoropurine 2’‐deoxyriboside (**36**)	9	269	273	271	310
	adenosine‐5’‐monophosphate (**37**)	13	259	268	267	310
	guanosine‐5’‐monophosphate (**38**)	13	264	273	279	310
	inosine‐5’‐monophosphate (**39**)	13	252	262	263	320
	1,2,4‐triazole‐3‐carboxamide riboside (**40**)^[d]^	13	–^[e]^	–^[e]^	–^[e]^	–^[e]^

[a] See Figure S1 for the structures of all compounds. [b] pH 9 was generally achieved in 50 mM Tris/NaOH buffer, pH 10 in 100 mM glycine/NaOH buffer, pH 13 in 100 mM NaOH, pH 13.3 in 200 mM NaOH and pH 13.7 in 500 mM NaOH. [c] From the original report.^14^ [d] Ribavirin. [e] Both *λ*_max_ values are at <250 nm, and there is no isosbestic point of base cleavage. Note that reaction monitoring can still be performed by single‐ or multi‐wavelength detection, but normalization to the isosbestic point of base cleavage is not possible for this substrate.

## Dealing with background

**Figure 2:**
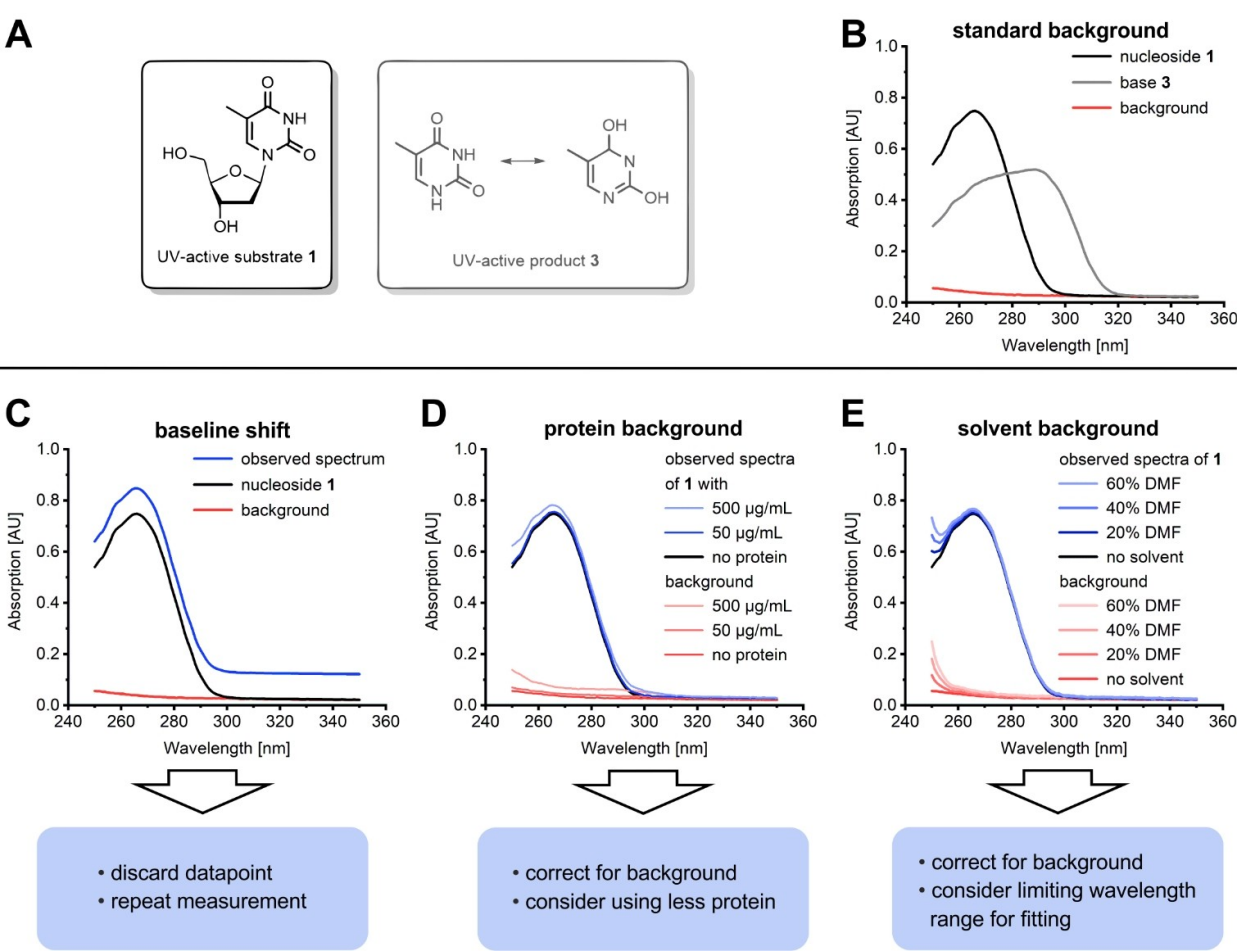
Common background signals. A) Examplary UV‐active reactants **1** and **3**. B) Standard background observed from the absorption of the 96‐well plate filled with water or aqueous NaOH. Signals for **1** and **3** represent typical signal intensities observed for reactions with 2 mM nucleoside substrate and a dilution factor of 15 during sampling. C) Baseline shift observed in approximately 1–2 % of measurements. D) Background observed in reactions with significant protein content. Purified *Escherichia coli* thymidine phosphorylase was used to recreate typical protein backgrounds by 15‐fold dilution in 100 mM NaOH. Note that some proteins can cause significantly more or less background. E) Representative background observed with some organic solvents. DMF was diluted tenfold in 100 mM NaOH to record the background signals.

The most common obstacle with the presented method is background absorption. Different types of background absorption are typically observed and need to be addressed individually (Figure 2). Please note that while we use the phosphorolysis of thymidine (**1**, Figure 2A) as an example reaction in this manuscript, the same principles translate to all nucleosides and nucleotides and can be applied in the same manner.

An occasional and unavoidable type of background signal is atypical UV absorption of the multiwell plate or of particles. A typical background signal is in the range of 0.03 absorption units (AU) at 300 nm and curves up towards slightly higher intensities at 250 nm (Figure 2B). This background is very well reproducible and easily adjusted for, as described in our original method. However, on average in approximately 1–2 % of all measurements, we observed increased absorption across the entire spectrum apparent as a distinct baseline shift (Figure 2C). This results in an inability to obtain accurate fits without manual spectral processing (which we choose to explicitly abstain from), as in these cases the baseline can be shifted >0.10 AU. The straightforward solution in this case is remeasurement of the sample in a different well, which generally resolves the problem.

We did not mention correction for protein background in our initial report. That is because in most cases enzyme background absorption in the considered range of 250–350 nm is quasi indetectable when using protein concentrations of 50 μg/mL or less in the reaction mixture that is sampled. In selected instances where either higher concentrations and/or especially UV‐active enzymes are used, appropriate background correction of the experimental sample may be necessary (Figure 2D). This can easily be carried out with a spectrum of a suitably diluted sample of the enzyme. Note that one may keep using the previously obtained reference spectra for the substrate and product without any further alterations as those are already corrected for their respective background.

Background absorption by UV‐active components in the reaction mixture needs to be addressed on a case‐to‐case basis. Generally, all common buffers, protein stabilizing agents or even artifacts from protein purification such as imidazole do not represent any challenge to the method and allow straightforward use of the standard procedure without any additional background correction. When using some organic cosolvents, however, we noticed significant background absorption in the lower wavelength region. Whereas alcohols including methanol, ethanol, isopropanol, ethylene glycol and glycerol may be used without alterations to the method (see Supporting Information for details), solvents like dimethyl sulfoxide (DMSO) and dimethyl formamide (DMF) create background signals that need to be accounted for (Figure 2E). In these cases we found success employing background spectra that reflect the specific content of UV‐active solvent in the sample and, if appropriate, limiting the wavelength range for fitting of pyrimidine UV absorption spectra to the information‐rich tail region (i. e., 265–295 nm for uridine, **4**). We even found success when using especially UV‐active reaction components, such as dithiothreitol (DTT), that proved problematic in some instances, by selecting appropriate substrates and selectively limiting the fitting range (see the Supporting Information for details and Figure S3 for an example).

In rare cases, we observed elevated baselines when using very concentrated buffers for dilution and spectral measurement. Again, appropriate background spectra to correct for this shift succeeded in resolving this issue.

Nonetheless, it should be stressed that background correction does not always lead to accurate and reliable data. We experienced serious difficulties to correct for noise in instances where the background signal intensity is comparable to the signal intensity of the nucleoside‐nucleobase pair under investigation and directly and/or completely overlaps with this signal (i. e., when examining the effect of enzyme inhibitors in stoichiometric quantities). In principle, background subtraction from the experimental spectrum is still possible and yields a processed spectrum that can be fitted, but fit quality and, consequently, accuracy of this approach suffered tremendously. We ascribed this to the fact that the signal intensity of the dynamic analytes (substrate and product) is largely irrelevant as those values are normalized to the isosbestic point and considered only in relation to one another, but background signals from most sources are absolute quantities and thus vary with and are very sensitive to pipetting accuracy. As a rule of thumb, we recommend our method for cases where background absorption does not exceed 20 % of the relevant signals (i. e., signal‐to‐noise ratio should remain >5).

## Reactant Instability

A critical factor for any method is stability and detectability of the analytes. While all nucleosides and nucleobases in our original report displayed excellent stability towards the quenching and analysis conditions, we noted some issues within the extended substrate range. Fluorinated purine nucleosides **22** and **23** were found to be quite sensitive to alkaline conditions as these nucleosides underwent a temperature‐ and base‐promoted side reaction (Figure S2), presumably by 5’−OH attack at the purine ring. We were able to bypass this issue by quick sample processing avoiding any unnecessary storage. At room temperature and pH 13, compounds **22** and **23** remained stable enough for analysis for at least 10 min.

## Further applications of the method

While the original protocol has proven to be a robust and versatile method, we have used spectral unmixing‐based reaction monitoring in instances that deviated from the original conditions. Some applications that necessitated adjusted sample treatment are worth mentioning.

In our earlier report we have used purified protein in all reactions.^14^ The subsequently published applications also featured pure protein in all instances.^12^ However, the use of unpurified protein, for example, in the form of crude cell lysate, is highly desirable for screening of mutants or whole‐cell reactions. We were pleased to find that even crude protein preparations (as lysed cells or cell‐free extract) permitted the use of spectral unmixing‐based reaction monitoring, if appropriate background correction is considered and the background signal remains within a manageable range (see above and the Supporting Information). In cases where heterogenous reactions were applied, centrifugation of the quenched alkaline samples prior to analysis was necessary and successfully reduced background noise and baseline shifts caused by particles.

Conveniently, spectral shifting of the UV absorption spectra of the free nucleobases doesn't always require application of a strong base. Selected nucleoside‐nucleobase pairs feature a marked spectral shift and stable spectra in a pH region easily accessible by established buffer systems (e. g., **33**–**36**, Table 1). This also presents an opportunity to monitor live reactions, either by applying a continuous assay or discontinuously monitoring very slow reactions by diluting samples of the reaction mixture in appropriate buffer (see the Supporting Information for details).

Some nucleosides precluded application of the original protocol that involves quenching of reaction samples in aqueous NaOH. Chlorinated scaffolds **33**–**36** display remarkably dynamic UV absorption spectra at pH values above 11 (Figure S4) and we were unable to obtain reproducible spectral fits using alkaline quenching. Fortunately, this issue could be resolved by employing organic solvents like methanol as an alternative quenching medium and subsequently adjusting the pH value to 9 for analysis (see the Supporting Information for details). A similar methodology featuring a different buffer system succeeded for the trifluorinated pyrimidine **15** (Table 1, Figure S6).

These examples only present a snapshot of the diverse applications of spectral unmixing‐based reaction monitoring of transformations between nucleosides and nucleobases that one may envision. Nonetheless, we are confident that the lessons learned thus far will translate well to other scenarios, reaction systems, enzymes and applications where similar issues might be encountered.

## Conclusion

Spectral unmixing presents a powerful tool for the efficient reaction monitoring of nucleobase cleavage reactions, with nucleoside phosphorolysis representing a highly relevant example. Spectral unmixing of UV absorption spectra conveniently allows for increased sample throughput compared to other methods and doesn't require expensive equipment or reagents. We have employed this method extensively and demonstrated its precision, versatility, robustness and ease of handling. This report extends the range of established substrates and discusses common problems and notable modifications to the original protocol. Reference spectra for all substrates and nucleobases listed in this article^36^ as well as our Python code used for spectral unmixing^37^, ^38^ can be obtained from an external online repository and we are happy to assist with their use. While specific scenarios may require evaluation and troubleshooting on a case‐to‐case basis, the strategies discussed herein will facilitate the straightforward and versatile application of this method.

## Experimental Section

All chemicals were purchased from Sigma Aldrich, TCI, Carl Roth, Carbosynth or BioNukleo GmbH at the highest available quality and used without prior purification. Solutions of all compounds were prepared in water deionized to 18.2 MW⋅cm with a Werner water purification system. NaOH solutions were prepared with deionized water. Enzymatic reactions were typically prepared from stock solutions of substrates and buffer and started via the addition of enzyme. Various NPs were used, including among others Y01, Y02, N01 and N02 from BioNukleo GmbH, *E. coli* uridine and thymidine phosphorylase and purine NP and *Bacillus subtilis* pyrimidine NP, as described previously.^12^ UV absorption spectra were recorded on a BioTek PowerWave HT plate reader, using UV/Vis‐transparent 96‐well plates (UV‐STAR F‐Bottom #655801, Greiner Bio‐One). Spectral processing, unmixing and data generation were performed as described previously^14^ with software freely available online.^37^, ^38^ All data presented in this report and the Supporting Information are freely available from an external online repository.^36^

Reference spectra for pure compounds were prepared from 2 mM solutions by 10‐ to 20‐fold dilution in aqueous NaOH. Reactions with purine or pyrimidine nucleosides were typically performed with either 1 or 2 mM of UV‐active compounds. From the 2 mM reactions, 20 μL (purines) or 30 μL (pyrimidines) were withdrawn and quenched in NaOH to give a final volume of 500 μL. From the 1 mM reactions, twice as much sample volume was withdrawn and treated analogously. Note that exact adherence to these volumes is not necessary, as normalization to the isosbestic point accounts for differences in signal intensity. For halogenated nucleosides **33**–**36**, typically, samples of 20 μL were withdrawn and either diluted in 50 mM Tris/NaOH buffer (pH 9) to a volume of 500 μL or quenched in an equal volume of MeOH or *i*PrOH before dilution in 50 mM Tris/NaOH buffer to give a final volume of 500 μL. Similarly, the fluorinated pyrimidine **15** was sampled by quenching in *i*PrOH or MeOH followed by dilution with 100 mM glycine/NaOH buffer (pH 10) to a final volume of 500 μL. Subsequently, 200 μL of the diluted alkaline samples were transferred to wells of a UV/Vis‐transparent 96‐well plate to record the UV absorption spectra. All spectra were recorded from 250–350 nm in steps of 1 nm. For exact sampling procedures and example reactions, please see our earlier reports^12^, ^14^ and the Supporting Information.

## Supporting information

As a service to our authors and readers, this journal provides supporting information supplied by the authors. Such materials are peer reviewed and may be re‐organized for online delivery, but are not copy‐edited or typeset. Technical support issues arising from supporting information (other than missing files) should be addressed to the authors.

Supplementary
