# Supplementary material for: Spectral Unmixing‐Based Reaction Monitoring of Transformations between Nucleosides and Nucleobases
Source: Chembiochem. 2020 Jun 18;21(18):2604–10. doi: 10.1002/cbic.202000204 (PMC7540295; doi:10.1002/cbic.202000204)
Supplement: Supplementary file 1 — Supplementary [file CBIC-21-2604-s001.pdf]

ChemBioChem

Supporting Information

Spectral Unmixing-Based Reaction Monitoring of Transformations between Nucleosides and Nucleobases**

Felix Kaspar,* Robert T. Giessmann, Sarah Westarp, Katja F. Hellendahl, Niels Krausch, Isabel Thiele, Miriam C. Walczak, Peter Neubauer, and Anke Wagner*

Author Contributions

F.K. Conceptualization:Lead; Data curation:Equal; Formal analysis:Equal; Investigation:Lead; Methodology:Equal; Project administration:Lead; Supervision:Lead; Visualization:Lead; Writing - Original Draft:Lead; Writing - Review & Editing:Lead
R.G. Data curation:Lead; Formal analysis:Equal; Funding acquisition:Equal; Methodology:Equal; Resources:Equal; Software:Lead; Writing - Review & Editing:Supporting
S.W. Investigation:Supporting; Writing - Review & Editing:Supporting
K.H. Investigation:Supporting; Writing - Review & Editing:Supporting
N.K. Formal analysis:Equal; Methodology:Supporting; Software:Equal; Writing - Review & Editing:Supporting
I.T. Investigation:Supporting; Writing - Review & Editing:Supporting
M.W. Investigation:Supporting; Writing - Review & Editing:Supporting
P.N. Funding acquisition:Equal; Resources:Equal; Writing - Review & Editing:Supporting
A.W. Funding acquisition:Equal; Resources:Equal; Writing - Review & Editing:Supporting

1
Figure S1. Nucleosides and nucleotides in this study 2
Figure S2. Degradation of fluorinated nucleoside **22** 2
Figure S3. UV absorption spectra of nucleosides and dithiothreitol (DTT) 3
Figure S4. Dynamic UV absorption spectra of chlorinated purine scaffold **33** 3
Figure S5. Spectral unmixing of mixtures of **33** and its free nucleobase 4
Figure S6. pH-dependent UV absorption spectra of 5-trifluoromethyluracil 4
Figure S7. Flowchart for experimental sampling procedure 5
A Note on the use of crude protein preparations 6
References 6

Author Contributions (with definitions as recommended by Brand *et al.*[1])

Conceptualization, F.K.; Data curation, F.K. and R.T.G.; Formal analysis, F.K., R.T.G. and N.K.; Funding acquisition, R.T.G., P.N. and A.W.; Investigation, F.K., S.W., K.F.H., I.T. and C.W.; Methodology, F.K., R.T.G. and N.K.; Project administration, F.K.; Resources, R.T.G., A.W. and P.N.; Software, R.T.G. and N.K.; Supervision, F.K.; Validation, - ; Visualization, F.K.; Writing—original draft, F.K.; Writing—review & editing, F.K., R.T.G., S.W., K.F.H., N.K., I.T., C.W. P.N. and A.W.

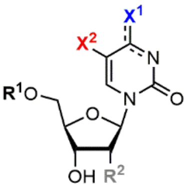

Pyrimidines

Compound	R^1	R^2	X^1	X^2
Uridine (**4**)	H	OH	O	H
2'-Deoxyuridine (**5**)	H	H	O	H
5-Methyluridine (**6**)	H	OH	O	Me
Thymidine (**1**)	H	H	O	Me
5-Fluorouridine (**7**)	H	OH	O	F
2'-Deoxy-5-fluorouridine (**8**)	H	H	O	F
5-Bromouridine (**9**)	H	OH	O	Br
5-Bromo-2'-deoxyuridine (**10**)	H	H	O	Br
5-Iodouridine (**11**)	H	OH	O	I
2'-Deoxy-5-iodouridine (**12**)	H	H	O	I
5-Ethynyluridine (**13**)	H	OH	O	ethynyl
2'-Deoxy-5-ethynyluridine (**14**)	H	H	O	ethynyl
5-Trifluorouridine (**15**)	H	OH	O	CF_3
Cytidine (**16**)	H	OH	NH_2	H
2'-Deoxycytidine (**17**)	H	H	NH_2	H
Uridine-5'-monophosphate (**18**)	PO_3^{2-}	OH	O	H
Cytidine-5'-monophosphate (**19**)	PO_3^{2-}	OH	NH_2	H

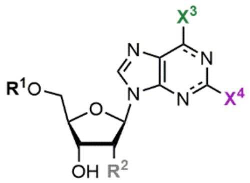

Purines

Compound	R^1	R^2	X^3	X^4
Adenosine (**20**)	H	OH	NH_2	H
2'-Deoxyadenosine (**21**)	H	H	NH_2	H
2-Fluoroadenosine (**22**)	H	OH	NH_2	F
2'-Deoxy-5-fluoroadenosine (**23**)	H	H	NH_2	F
2-Chloroadenosine (**24**)	H	OH	NH_2	Cl
2-Chloro-2'-deoxyadenosine (**25**)	H	H	NH_2	Cl
2-Aminoadenosine (**26**)	H	OH	NH_2	NH_2
2-Amino-2'-deoxyadenosine (**27**)	H	H	NH_2	NH_2
Guanosine (**28**)	H	OH	O	NH_2
2'-Deoxyguanosine (**29**)	H	H	O	NH_2
Inosine (**30**)	H	OH	O	H
2'-Deoxyinosine (**31**)	H	H	O	H
Xanthosine (**32**)	H	OH	O	O
2,6-Dichloropurine riboside (**33**)	H	OH	Cl	Cl
2,6-Dichloro 2'-deoxyriboside (**34**)	H	H	Cl	Cl
6-Chloro-2-fluoropurine riboside (**35**)	H	OH	Cl	F
6-Chloro-2-fluoropurine 2'-deoxyriboside (**36**)	H	H	Cl	F
Adenosine-5'-monophosphate (**37**)	PO_3^{2-}	OH	NH_2	H
Guanosine-5'-monophosphate (**38**)	PO_3^{2-}	OH	O	NH_2
Inosine-5'-monophosphate (**39**)	PO_3^{2-}	OH	O	H

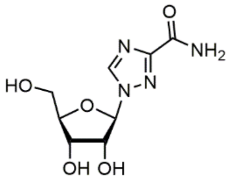

1,2,4-Triazole-3-carboxamide riboside (ribavirin, **40**)

Figure S1. Nucleosides and nucleotides in this study.

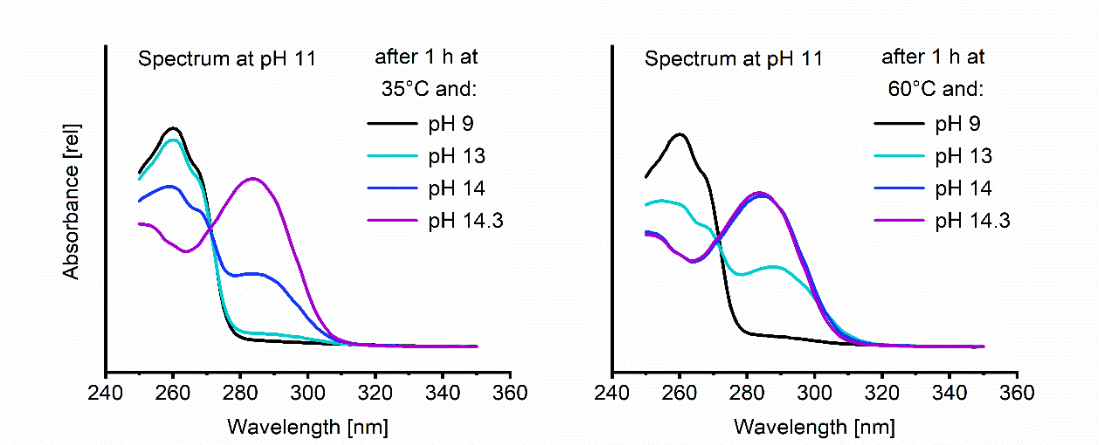

Figure S2. Degradation of fluorinated nucleoside **22**. 1 mM solutions of **22** were kept in either 50 mM glycine buffer (pH 9), 100 mM NaOH (pH 13), 1 M NaOH (pH 14) or 5 mM NaOH (pH 14.3) at 35 °C or 60 °C for 1 h. Samples of 40 µL were diluted in 460 µL 1 M H_2HPO_4 (pH 11) for measurement of UV/Vis spectra. A compound-to-compound conversion appears likely given the isosbestic point at 275 nm. Both base and temperature accelerated the apparent reaction. A similar behavior was observed for compound **23**.

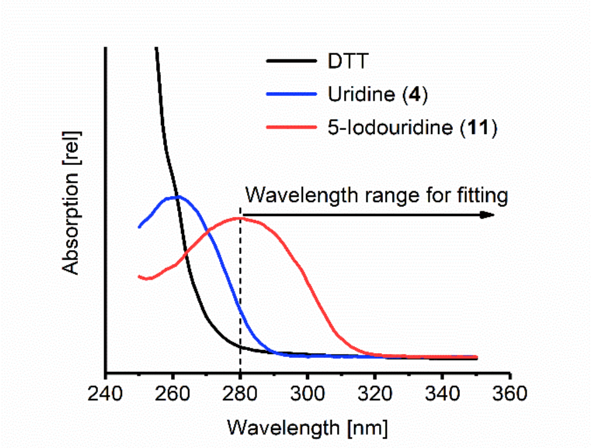

Figure S3. UV absorption spectra of nucleosides and dithiothreitol (DTT). With 5 mM DTT, the spectrum of DTT largely completely overlaps with that of 1 mM uridine (**4**, left), but the spectra of other nucleosides such as 5-iodouridine (**11**, right) can still be deconvoluted well from the reaction mixtures. A typical DTT spectrum (alkaline dilution factor of 9) and reference spectra for **4** (in 100 mM NaOH) and **11** (in 200 mM NaOH) are shown. All spectra in this figure are background corrected for multiwell plate absorption.

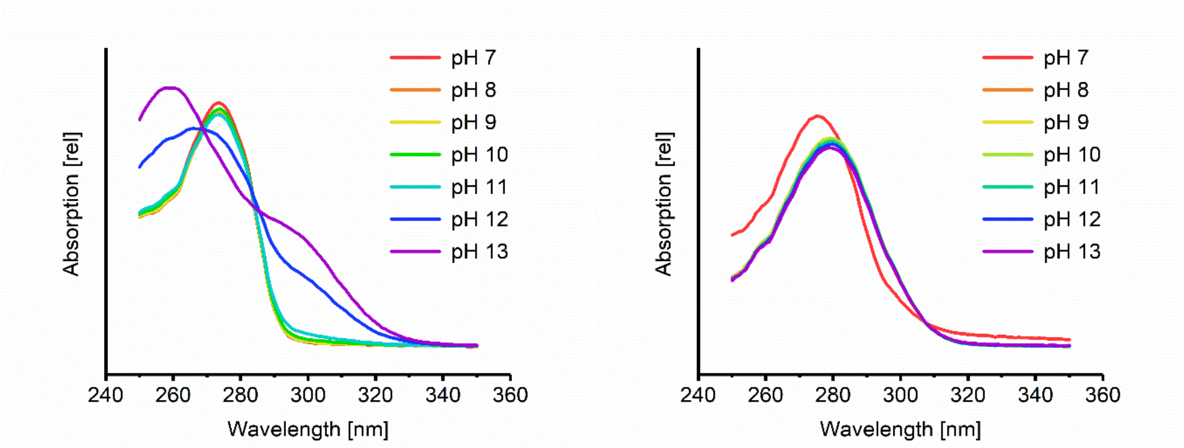

Figure S4. Dynamic UV absorption spectra of chlorinated purine scaffold **33**. Ribosyl nucleoside **33** (left) displays marked spectral "wobbling" above pH 11, compared to its free base (right). At pH 9 (yellow line), both compounds provide stable and reproducible spectra that allow robust unmixing.

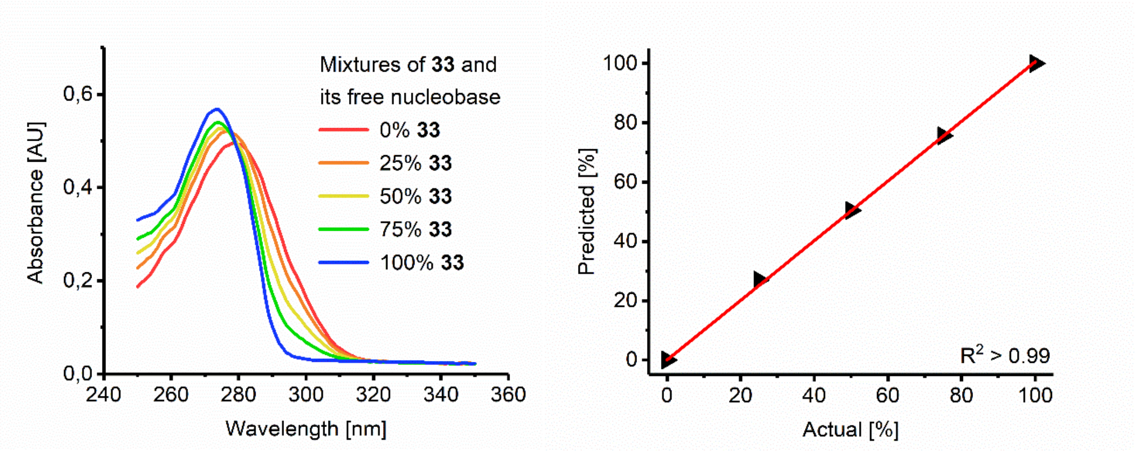

Figure S5. Spectral unmixing of mixtures of **33** and its free nucleobase. Mixtures of **33** with its base deliver stable and reproducible spectra at pH 9 with an isosbestic point of base cleavage at 278 nm (left; non-normalized raw data is shown), which allows for accurate unmixing under these conditions with reference spectra obtained at pH 9 (right).

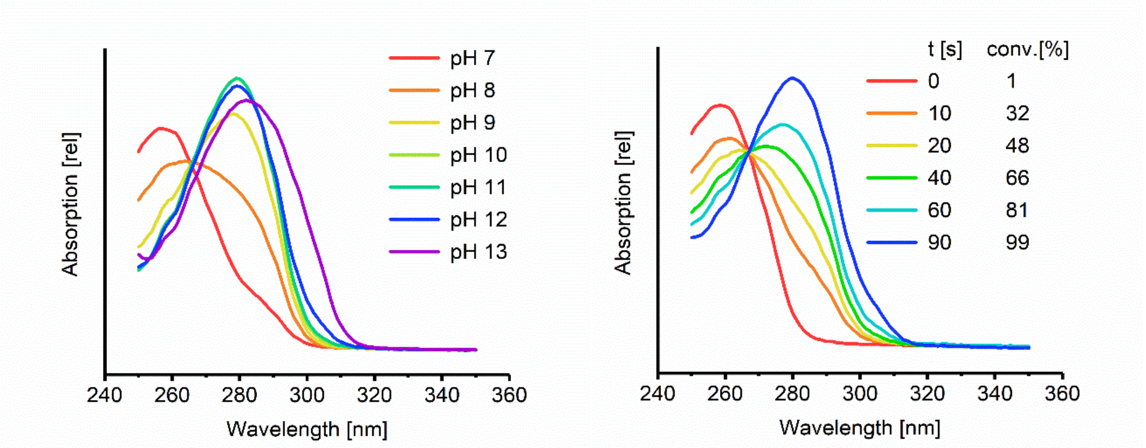

Figure S6. pH-dependent UV absorption spectra of 5-trifluoromethyluracil (the free base of **15**, left) and experimental spectra obtained from a reaction quenched in iPrOH and diluted in 100 mM glycine/NaOH buffer (pH 10) which allowed spectral unmixing-based reaction monitoring (right, spectra were normalized to the isosbestic point at 267 nm). The reaction was performed with 1 mM **15**, 50 mM phosphate and 10 µg·mL^{-1} pyrimidine nucleoside phosphorylase Y07 (BioNukleo GmbH, Berlin, Germany) in 100 mM glycine buffer at pH 9 and 80 °C. The reaction samples (50 µL) were quenched in 50 µL iPrOH and subsequently diluted with 400 µL 100 mM glycine/NaOH buffer (pH 10). Also see Figure S7.

Experimental Workflow

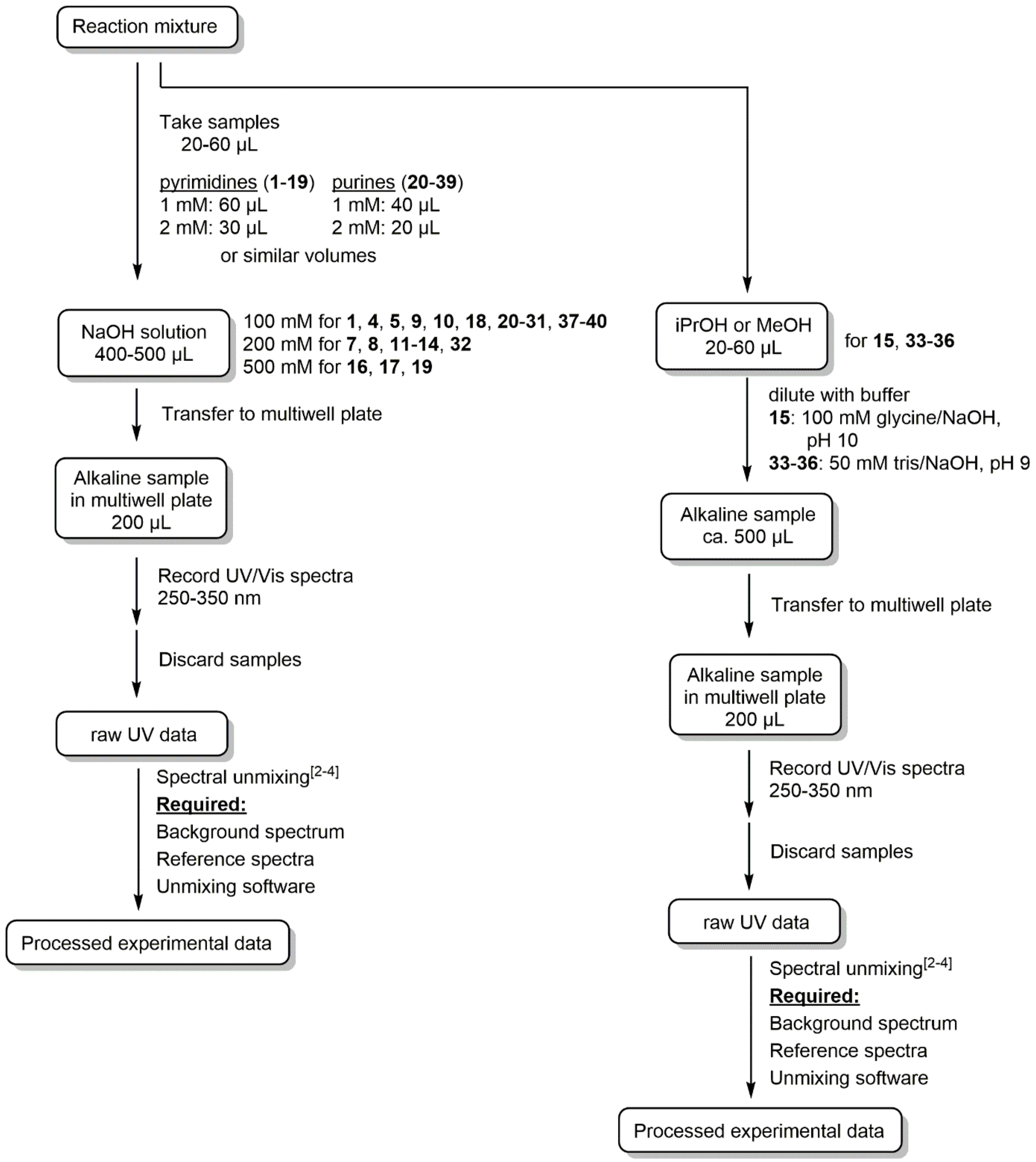

Figure S7. Flowchart for experimental sampling procedure. The process for sampling and sample processing is described. Please note that exact adherence to the suggested volumes is not required and, thus, purely optional. For the spectral unmixing procedure, please see the main text of the publication, the original report[2], as well as the associated external supplementary material[3] and the spectral unmixing Python code.[4] To facilitate the use of the method, reference spectra for substrates **1** and **4**—**40** can obtained from an external online repository.[3]

A Note on the use of crude protein preparations

As mentioned in the main text, crude protein preparations (either as cell lysate or cell-free extracts) do permit the use of spectral unmixing-based reaction monitoring. However, these applications may need to be assessed on a case-to-case basis, depending on the level of background absorption and sample homogeneity. Unfortunately, there is not one size fits-all and it is not possible to give conclusive guidelines on this issue since crude protein preparations vary from enzyme to enzyme and sometimes batch to batch. Generally, applications that do not require high accuracy are quite tolerant to background absorption by crude protein preparations with pyrimidines being more robust than purines. Thus, screening efforts tolerate significant background signals (up to a certain point; we recommend a signal-to-noise ratio of > 5) but applications that strive for high fit quality and accuracy will inevitably profit from a careful management of background absorption. The latter can be addressed by several routes. Reactions with cell lysates require centrifugation to remove particles prior to spectral analysis in most cases, although this may not be required in every instance. Background signal heterogeneity between different crude protein preparations can be addressed by obtaining reactant-free background signals from every protein preparation under analysis (which may prove unfeasible for screening, but mandatory for high accuracy). High background signals from these preparations may require using less protein in the reactions and running these for a longer time to achieve an equivalent level of conversion. Lastly, the application of purified proteins (in our experience a single affinity chromatography step is sufficient, but heat treatment is also enough for many thermostable proteins) is always desirable to achieve low background signals, high reproducibility, fit quality and accuracy. As mentioned above, analysis of reactions with pyrimidine nucleosides is generally more robust than with purine nucleosides (owed to their UV absorption spectra), but we have also had success employing spectral unmixing to reactions with some purine nucleosides and cell-free extracts (please see the files “FEK180” and “FEK180_results” in the external supplementary information[3] for a reaction with 2-chloroadenosine and 2% cell-free extract of a thermostable purine nucleoside phosphorylase).

References

[1] A. Brand, L. Allen, M. Altman, M. Hlava, J. Scott, *Learn. Publ.* **2015**, *28*, 151–155.

[2] F. Kaspar, R. T. Giessmann, N. Krausch, P. Neubauer, A. Wagner, M. Gimpel, Methods Protoc. **2019**, *2*, 60.

[3] F. Kaspar, **2020**, DOI 10.5281/zenodo.3716126.

[4] R. T. Giessmann, N. Krausch, **2019**, DOI 10.5281/zenodo.3243376.
